# Antithrombotic management in atrial fibrillation patients following percutaneous coronary intervention: A clinical review

**DOI:** 10.1002/joa3.13128

**Published:** 2024-08-08

**Authors:** Yuichi Saito, Yoshio Kobayashi

**Affiliations:** ^1^ Department of Cardiovascular Medicine Chiba University Graduate School of Medicine Chiba Japan

**Keywords:** antithrombotic therapy, atrial fibrillation, percutaneous coronary intervention

## Abstract

Patients with atrial fibrillation (AF) often develop acute coronary syndrome and undergo percutaneous coronary intervention (PCI), and vice versa. Acute coronary syndrome and PCI mandate the use of dual antiplatelet therapy, while oral anticoagulation is recommended in patients with AF to mitigate thromboembolic risks. Clinical evidence concerning antithrombotic treatment in patients with AF and PCI has been accumulated, but when combined, the therapeutic strategy becomes complex. Although triple therapy, a combination of oral anticoagulation with dual antiplatelet therapy, has been used for patients with AF undergoing PCI as an initial antithrombotic strategy, less intensive regimens may be associated with a lower rate of bleeding without an increased risk in thrombotic events. This narrative review article summarizes currently available evidence of antithrombotic therapy in patients with AF undergoing PCI.

## Graphical abstract

In patients with atrial fibrillation (AF) undergoing percutaneous coronary intervention (PCI), antithrombotic management becomes complex to balance ischemic and bleeding risks. In this context, the recent clinical evidence indicates the “less is more” concept in antiplatelet therapy and oral anticoagulation (OAC). In this review paper, the current understanding and future perspectives in antithrombotic therapy in this setting are summarized.

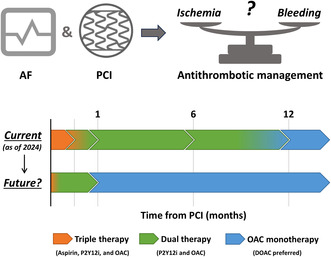

## 1 INTRODUCTION

Atrial fibrillation (AF) is a common cardiac rhythm disturbance leading to heart failure and thromboembolic events.^1^ The estimated prevalence of AF in Japan and East Asia was approximately 1000 cases per 100 000 population in 2020, projected to rise for the next decades globally.^1^ In patients with AF, concomitant coronary artery disease (CAD) is often present, and vice versa. About 20% of patients with AF reportedly develop acute coronary syndrome or undergo percutaneous coronary intervention (PCI).^2^ Although antithrombotic therapies are a cornerstone of the management of patients with AF and those undergoing PCI to prevent thrombotic and ischemic events, the therapeutic regimens become complex when both conditions coexist. Dual antiplatelet therapy (DAPT) is indicated, at least as an initial antiplatelet regimen, for patients undergoing PCI to mitigate the risk of myocardial infarction and stent thrombosis,^3^ while oral anticoagulation (OAC) is beneficial in reducing thromboembolic events (e.g. ischemic stroke) to a greater extent than DAPT in patients with AF.^4^ Triple antithrombotic therapy, a combination of DAPT plus an OAC, has been used in clinical practice but is associated with an increased risk of serious bleeding events.^5^ According to several randomized control trial (RCT) results, guidelines have been updated, showcasing the “less is more” concept.^6^ This narrative review article briefly summarizes the current evidence concerning antithrombotic therapy in patients with AF undergoing PCI.

## 2 CLINICAL TRIAL RESULTS

Since the clinical efficacy of DAPT as compared with OAC was established in RCTs in the late 1990s, DAPT consisting of aspirin and a P2Y12 inhibitor has been the cornerstone of antithrombotic management in patients undergoing PCI,^3^ while OAC with vitamin K antagonist (VKA) was superior over DAPT in reducing ischemic events among patients with AF.^4^ In recent decades, direct oral anticoagulation (DOAC) has been shown to have a favorable risk–benefit profile as compared to VKA, particularly in the reduction of intracranial hemorrhage.^7^ Thus, a combination of DAPT with OAC may be “theoretically” necessary for patients with AF undergoing PCI. Yet, the clinical effectiveness of this intensive antithrombotic strategy has not been well‐proven. The term “triple (antithrombotic) therapy” indicates a combination of OAC (VKA or DOAC) plus DAPT (aspirin and a P2Y_12_ inhibitor), while “dual (double) therapy” includes an OAC with single antiplatelet therapy (SAPT) (aspirin or a P2Y_12_ inhibitor).^8^ In addition, OAC monotherapy is a regimen of single OAC (VKA or DOAC) with no antiplatelet agents.

To date, several RCTs have shown that triple therapy is associated with an increased risk of major bleeding events in patients undergoing PCI with an OAC indication (Table 1).^9^, ^10^, ^11^, ^12^, ^13^, ^14^, ^15^, ^16^ A meta‐analysis confirmed that DOAC‐based dual (double) therapy after periprocedural triple therapy for 3 to 14 days resulted in a lower risk of major and intracranial hemorrhages as compared with VKA‐based triple therapy for 1 to 12 months, although the less potent antithrombotic regimen (i.e. dual therapy) was probably associated with a higher risk of stent thrombosis.^17^ However, dual therapy may be reasonable because of the relatively low event risk of stent thrombosis rather than major bleeding in this patient population. Of note, Pivotal RCTs in Table 1 such as the WOEST, PIONEER AF‐PCI, RE‐DUAL PCI, AUGUSTUS, and ENTRUST‐AF PCI trials randomized patients to either the dual or triple antithrombotic regimen 3 to 14 days after PCI, during which triple therapy was applied even to the experimental dual therapy group. Thus, whether triple therapy, aspirin in particular, can be safely omitted at the time of PCI remains unclear. Another recent meta‐analysis reinforced that periprocedural DAPT was better in terms of bleeding outcomes as compared to short‐ (4–6 weeks) or long‐term (≥3 months) DAPT on top of OAC in patients undergoing PCI with an indication for anticoagulation.^18^ Beyond 12 months after PCI, lifelong OAC with no antiplatelet therapy has been recommended in the guidelines according to the historical data with VKA. In this context, two RCTs from Japan, the OAC‐ALONE and AFIRE trials, shed light on the evidence gap (Table 2). The OAC‐ALONE was the first RCT that included patients with AF and stable CAD beyond 1 year after coronary stenting.^19^ Although this study was prematurely terminated as a result of slow enrollment and resulted in insufficient statistical power, a signal of less frequent major bleeding events in the OAC monotherapy group than in the combined OAC plus SAPT group was found.^19^ The subsequent AFIRE trial demonstrated that rivaroxaban monotherapy was non‐inferior (and indeed superior) to the combined antithrombotic therapy of rivaroxaban plus SAPT in both ischemic and bleeding endpoints in patients with AF who had undergone PCI or coronary artery bypass grafting >1 year earlier or who had angiographically confirmed CAD.^20^ Although the underlying mechanism of fewer ischemic events in the rivaroxaban monotherapy group in the trial is uncertain, a sub‐analysis from the AFIRE showed that ischemic outcomes were likely to occur soon after a major bleeding event, which were presumably associated with a decreased threshold for myocardial ischemia and heart failure, potential harm of red blood cell transfusions, and discontinuation of antithrombotic therapy following bleedings.^21^ Thus, it is conceivable that OAC monotherapy beyond 1 year after PCI should be a default antithrombotic regimen in patients with AF and PCI. Probably because of recent advances in medical therapy and PCI technologies,^22^, ^23^, ^24^, ^25^, ^26^, ^27^, ^28^, ^29^, ^30^, ^31^, ^32^, ^33^, ^34^, ^35^, ^36^, ^37^, ^38^, ^39^, ^40^, ^41^, ^42^, ^43^, ^44^, ^45^, ^46^, ^47^, ^48^, ^49^, ^50^, ^51^, ^52^, ^53^, ^54^, ^55^, ^56^, ^57^, ^58^, ^59^, ^60^, ^61^, ^62^, ^63^ ischemic and thrombotic risks after interventional procedures have been declined, while bleeding events have been increased,^64^ leading to the “less is more” concept in antithrombotic regimens in the current era.

**TABLE 1:** Key randomized trials of antithrombotic therapy in patients with indications for SAPT and OAC.

	Publication year	Sample size	AF	PCI	Abbreviated DAPT	Prolonged DAPT	Tested OAC	Results
WOEST^9^	2013	573	69%	100%	Periprocedural DAPT followed by clopidogrel	≥1 m (BMS) or ≥12 m (DES)	VKA	Dual therapy reduced bleeding and death than TT
ISAR‐TRIPLE^10^	2015	614	84%	100%	6 w DAPT followed by aspirin	6 m DAPT (aspirin + clopidogrel)	VKA	6 w TT was not superior to 6 m TT in net clinical outcomes
PIONEER AF‐PCI^11^	2016	2124	100%	100%	Periprocedural DAPT only in the DOAC arm	1–12 m only in the VKA arm	Rivaroxaban vs. VKA^c^	Dual therapy with low‐dose rivaroxaban reduced bleeding events than VKA‐TT
RE‐DUAL PCI^12^	2017	2725	100%	100%	Periprocedural DAPT only in the DOAC arm	1–3 m only in the VKA arm	Dabigatran versus VKA	Dual therapy with dabigatran reduced bleeding events than VKA‐TT
AUGUSTUS^13^	2019	4614	100%	76%	Periprocedural DAPT followed by P2Y12i	6 m DAPT (aspirin + P2Y12i)	Apixaban versus VKA	Dual therapy with apixaban reduced bleeding events than VKA‐TT
ENTRUST‐AF PCI^14^	2019	1506	100%	100%	Periprocedural DAPT only in the DOAC arm	1–12 m only in the VKA arm	Edoxaban versus VKA	Edoxaban‐based dual therapy was non‐inferior to VKA‐based TT
SAFE‐A^15^	2020	208^a^	100%	100%	1 m DAPT (aspirin + P2Y12i) followed by SAPT	6 m DAPT (aspirin + P2Y12i)	Apixaban	Bleedings were non‐significantly fewer in abbreviated DAPT group
MASTER‐DAPT^16^	2021	1666^b^	84%	100%	1 m DAPT (aspirin + P2Y12i) followed by SAPT	≥3 m DAPT (aspirin + P2Y12i) followed by SAPT	VKA or DOAC	Bleedings were non‐significantly fewer in abbreviated DAPT group

Abbreviations: AF, atrial fibrillation; DAPT, dual antiplatelet therapy; DOAC, direct oral anticoagulation; OAC, oral anticoagulation, P2Y12i, P2Y_12_ inhibitor; PCI, percutaneous coronary intervention; SAPT, single antiplatelet therapy; TT, triple therapy; VKA, vitamin K antagonist.

^a^ Prematurely terminated because of a slow enrollment.

^b^ OAC sub‐analysis.

^c^ Low‐dose (15 mg daily) and very low‐dose (5 mg daily) rivaroxaban.

**TABLE 2:** Key randomized trials of antithrombotic therapy in patients with indications for SAPT and OAC at a chronic phase.

	Publication year	Sample size	AF	PCI	Experimental arm	Control arm	Tested OAC	Results
OAC‐ALONE^19^	2018	690^a^	100%	100%	OAC monotherapy	OAC plus SAPT	VKA or DOAC	Non‐inferiority of OAC alone strategy was not established
AFIRE^20^	2019	2215	100%	71%	Rivaroxaban monotherapy	Rivaroxaban plus SAPT	Rivaroxaban	Rivaroxaban monotherapy reduced ischemic and bleeding events

Abbreviations: AF, atrial fibrillation; DOAC, direct oral anticoagulation; OAC, oral anticoagulation, PCI, percutaneous coronary intervention; SAPT, single antiplatelet therapy; VKA, vitamin K antagonist.

^a^ Prematurely terminated because of a slow enrollment.

## 3 GUIDELINE RECOMMENDATIONS AND FUTURE PERSPECTIVES

**FIGURE 1:**
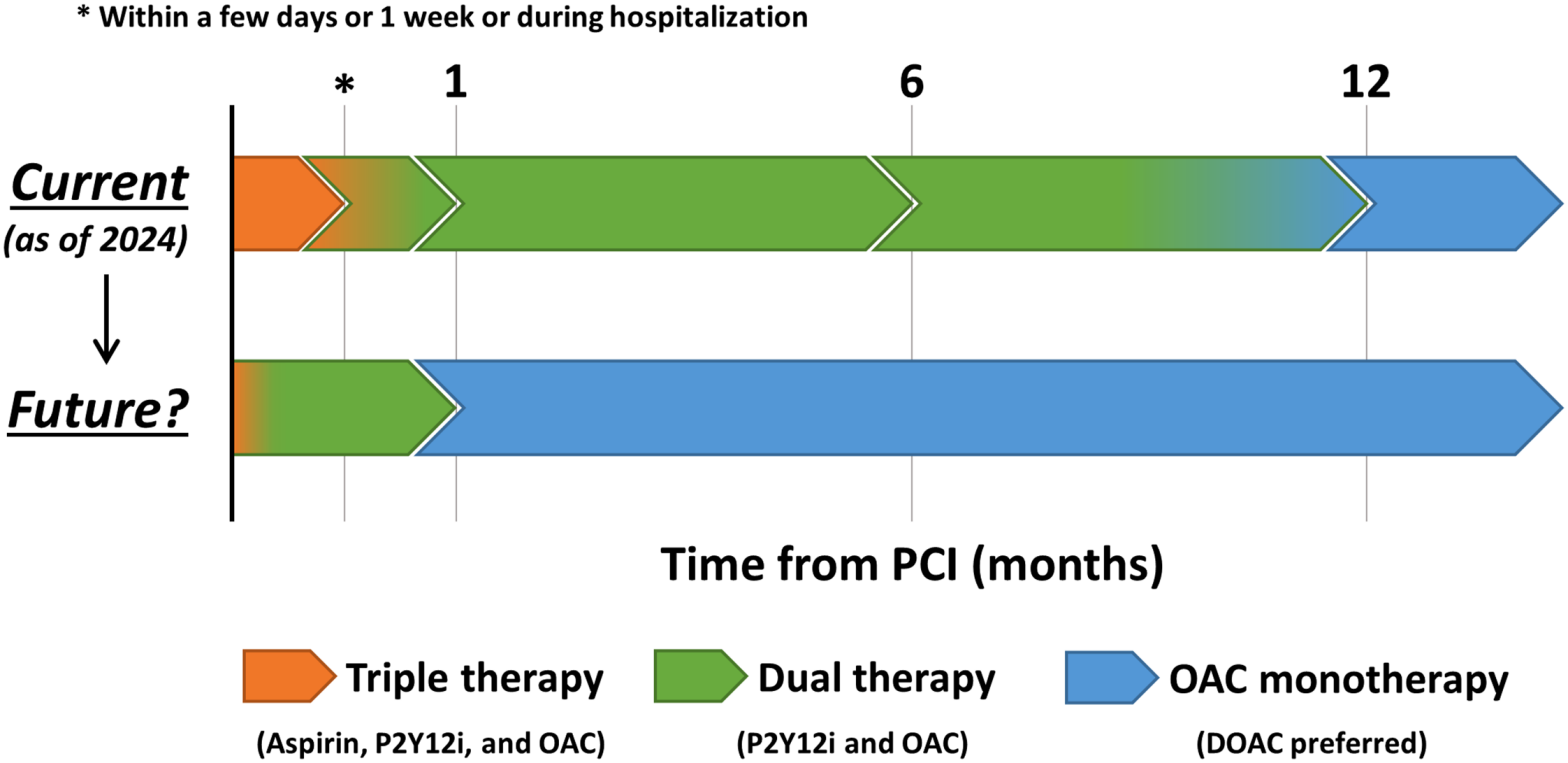
Conceptual algorithm of current and future antithrombotic therapy for patients with atrial fibrillation undergoing PCI. There is considerable overlap among risk factors associated with ischemic and bleeding outcomes, and an antithrombotic regimen should be balanced depending of individual ischemic and bleeding risks. Current guidelines and consensus documents recommend triple therapy (aspirin, a P2Y12 inhibitor, and OAC) for 1 to 30 days after PCI in patients with atrial fibrillation, followed by dual therapy (OAC plus a P2Y12 inhibitor) for (6 to) 12 months and OAC monotherapy thereafter. The potential future antithrombotic regimen includes no or 1‐day DAPT with DOAC, followed by dual therapy with a P2Y12 inhibitor plus DOAC for only 1 month, and DOAC monotherapy thereafter. A P2Y12 inhibitor is preferred as single antiplatelet therapy. DOAC may be better than vitamin K antagonist in this setting. DOAC, direct oral anticoagulation; OAC, oral anticoagulation; PCI, percutaneous coronary intervention.

According to the clinical evidence, recent guidelines and consensus documents recommend triple therapy for 1 to 30 days after PCI in patients with AF, followed by dual therapy (OAC plus a P2Y12 inhibitor) for up to 1 year and OAC monotherapy thereafter (Figure 1).^8^, ^65^, ^66^, ^67^, ^68^, ^69^ Discontinuation of aspirin within 7 days or at discharge is indicated in the recommendations, although triple therapy for up to 30 days is allowed based on individual ischemic and bleeding risks in AF patients undergoing PCI.^8^ In terms of intraprocedural anticoagulation in patients undergoing PCI who have an indication for OAC, activated clotting time >250 s with the use of unfractionated heparin during PCI is recommended.^70^ Recent trials have demonstrated that SAPT with a P2Y12 inhibitor rather than aspirin was associated with lower ischemic events after PCI,^71^, ^72^ and a P2Y12 inhibitor may be preferable over aspirin as a part of dual therapy with OAC.^69^ Additionally, DOAC is recommended over VKA to reduce intracranial hemorrhage.^8^, ^69^ Lifelong OAC monotherapy is indicated 1 year after PCI, but if the thrombotic risk is low, OAC alone beyond 6 months may be considered.^8^ The current recommendations are summarized in Figure 1. Of note, accurate ischemic and bleeding risk stratification is relevant in clinical decision‐making in antithrombotic therapy in patients undergoing PCI with AF, although no risk‐scoring systems have been established in this context. From a PCI perspective, the criteria of Academic Research Consortium for High Bleeding Risk (ARC‐HBR) are a guideline‐recommended tool and used globally for the bleeding risk stratification, while the Japanese guidelines recommend domestically modified criteria of ARC‐HBR in patients undergoing PCI.^69^ However, whether antithrombotic management guided by the ARC‐HBR criteria improves clinical outcomes in patients undergoing PCI is unclear. In terms of ischemic risk stratification, the Japanese guidelines indicate some scoring‐systems including the PARIS and CREDO‐Kyoto thrombotic risk scores to guide antithrombotic regimens after PCI.^69^ Yet, these scoring‐systems may not be widely employed in daily clinical practice. At present, ischemic and bleeding risk stratification using such risk scores may be reasonable, but future studies are warranted.

Given that recent trials investigating “aspirin‐free” regimens from the beginning of PCI have shown favorable results of the novel antithrombotic strategy, aspirin as a part of triple therapy may be safely omitted, at least in a setting of low thrombotic risk.^73^, ^74^, ^75^ Currently, aspirin‐free SAPT with a P2Y12 inhibitor (clopidogrel, prasugrel, and ticagrelor) is being tested globally in patients with acute coronary syndrome, including the NEO‐MINDSET (NCT04360720), LEGACY (NCT05125276), and PREMIUM (NCT05709626) trials.^76^ If these studies unveil the clinical effectiveness of aspirin‐free SAPT in an acute setting, no DAPT strategy may become a guideline‐recommended antiplatelet regimen in patients undergoing PCI. In AF patients undergoing PCI, the MATRIX‐2 (NCT05955365) and OPTIMA‐AF (jRCTs051190053) trials are currently ongoing to evaluate the safety and efficacy of shorter duration of dual therapy consisting of SAPT with a P2Y12 inhibitor plus DOAC for 1 month, followed by DOAC monotherapy, as compared to 6‐ or 12‐month dual therapy.^76^, ^77^ Aspirin is discontinued immediately after PCI in the experimental arms in the MATRIX‐2 and OPTIMA‐AF trials,^76^, ^77^ suggesting the clinical feasibility of minimum duration of DAPT (i.e. only at the time of PCI) in patients with AF and an indication for OAC. These trial results will shape our understanding of antithrombotic therapy in AF patients undergoing PCI, potentially leading to the regimen of no or 1‐day DAPT with DOAC, followed by dual therapy with a P2Y12 inhibitor plus DOAC for 1 month, and DOAC monotherapy thereafter (Figure 1). Furthermore, it is conceivable that left atrial appendage occlusion and resection will also play a significant role in this field.^78^, ^79^, ^80^, ^81^

## 4 CONCLUSIONS

Although the antithrombotic regimen should be balanced depending on ischemic and bleeding risks, enthusiastic efforts on antithrombotic therapy in recent decades have established the “less is more” concept in AF patients undergoing PCI. Future investigations will provide better guidance for antithrombotic regimens and standards for this patient population.

## CONFLICT OF INTEREST STATEMENT

All conflicts of interest are declared by authors in the manuscript.

## DISCLOSURES

Yuichi Saito received lecture fees from Daiichi Sankyo. Yoshio Kobayashi received lecture fees from Abbott Medical Japan and Daiichi Sankyo and research grants from Abbott Medical Japan, Win International, Otsuka Pharmaceutical, Boehringer Ingelheim, Nipro, and Japan Lifeline.
